# Sulfadiazine hosted in MIL-53(Al) as a biocide topical delivery system

**DOI:** 10.1039/d0ra03636f

**Published:** 2020-07-07

**Authors:** Javier Águila-Rosas, Tomás Quirino-Barreda, Gerardo Leyva-Gómez, Eduardo González-Zamora, Ilich A. Ibarra, Enrique Lima

**Affiliations:** Laboratorio de Farmacia Molecular y Liberación Controlada, UAM-Xochimilco Calzada del Hueso 1100, Col. Villa Quietud CP 04960 CDMX Mexico; Facultad de Química, Universidad Nacional Autónoma de México Circuito Exterior s/n, Cd. Universitaria, Del. Coyoacán CP 04510 CDMX Mexico; Universidad Autónoma Metropolitana-Iztapalapa San Rafael Atlixco 186, Col. Vicentina, Iztapalapa Ciudad de México CP 09340 Mexico; Laboratorio de Fisicoquímica y Reactividad de Superficies (LaFReS), Instituto de Investigaciones en Materiales, Universidad Nacional Autónoma de México Circuito Exterior s/n, Cd. Universitaria, Del. Coyoacán CP 04510 CDMX Mexico argel@unam.mx lima@iim.unam.mx +52-55-5622-4595; Pharma View Consulting SC CDMX Mexico; Doctorado en Ciencias Biológicas y de la Salud, Universidad Autónoma Metropolitana, UAM-Xochimilco Calzada del Hueso 1100, Col. Villa Quietud CP 04960, CDMX Mexico

## Abstract

Sulfadiazine (SDZ), a bacteriostatic agent, was hosted in a metal–organic framework, specifically in MIL-53(Al) and modified-zinc MIL-53(Al,Zn). Materials were characterized structural, and texturally. Both hosts loaded sulfadiazine but they were differenced regarding the release of sulfadiazine. The presence of zinc plays a significant role to the modulation of sulfadiazine–MOF interactions. Release of sulfadiazine from sulfadiazine@MOFs was monitored *in vitro* and *ex vivo* conditions. A kinetic release model is proposed for *in vitro* sulfadiazine release. Remarkably, the materials did not show cytotoxicity against eukaryote cells.

## Abstract 2

Sulfadiazine (SDZ), a bacteriostatic agent, was hosted in MIL-53(Al) and modified-zinc MIL-53(Al,Zn).

## Graphical abstract

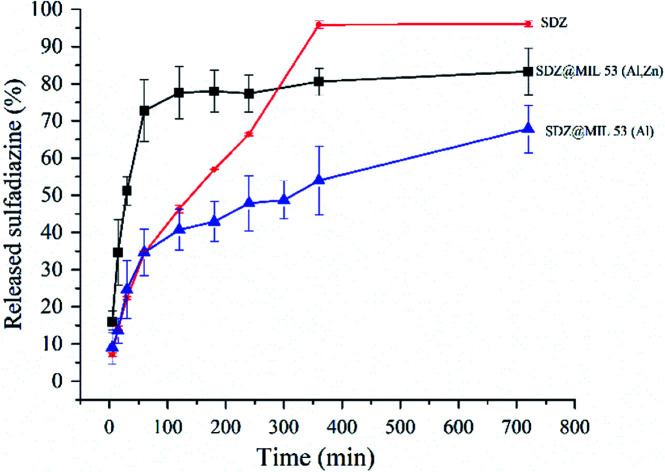

## Introduction

The drug delivery systems and bioactive molecules should be safe and efficient in a biological environment. It also should provide to the particular drug a great bioavailability and a prolongation of its effect.^1,2^ There are currently different types of releasers available, the most commonly used are micelles and liposomes and new promising candidates include carbon nanotubes, dendrimers, polymers, nanoparticles, among others.^3,4^

Recent materials proposed as drug delivers, porous coordination polymers (PCPs) are included, also known as MOFs which is the acronym of metal–organic frameworks.^5^ They are generally constructed of anionic organic ligands connected to metal cations of Mg^2+^, Zn^2+^, Al^3+^, Fe^3+^ and Cu^2+^. MOFs can be sized at micro or nanometric scale^6–9^ with an empty space of up to 90% of the total volume and high internal surfaces.^10,11^

The applications of MOFs in biological processes are more and more frequent; they are used as a diagnostic agent as well as a vector in the administration of drugs.^8,12–15^ The interest in these materials is mainly due to their pore size and large volume of pores available to transport biomolecules.

It is possible to develop biocompatible MOFs by synthesize them through hydro and solvothermal syntheses with low-boiling and non-toxic solvents. That way, it is possible to ensure that no traces of typically used toxic and non-biocompatible organic solvents (*e.g.*, DMF, DEF or THF), are found. It is worth mentioning that the synthesis of active ingredients or pharmaceutical formulations according to international pharmacopoeias, which prohibit use of these solvents or, in any case, the risk-benefit must be sustained when quantifying trace concentrations. Further, solvent residues in the structure represents a negative deviation in the results of cytotoxic tests and the pharmacological effect, resulting in a “green” synthesis for the acceptance of MOFs as novel drug delivery systems.^16–18^

MOFs such as MIL-53(Al), are excellent candidates as drug delivers.^19,20^ MIL-53(Cr and Fe) were used previously as drug delivery of model drug such as ibuprofen and keeping in mind release drug to bloodstream. In contrast, MOF containing Al^3+^ is suitable for local administration where systemic distribution is avoided. For example, this MOF material can be an interesting sulfadiazine transporter (SDZ). SDZ is a bacteriostatic agent, practically insoluble in aqueous solutions with low bioavailability and applied in high concentrations for prophylaxis and wound infections.

The structure of MIL-53(Al) shows three characteristic structural forms, the first one is identified as “as-synthesized (as)” which contains within the pores, the inclusion of terephthalic acid (BDC) as a result of its synthesis. After an appropriate heat treatment, MIL-53(Al) undergoes on a reversible structural change from a closed pore structure to an open pore structure, entitled “high temperature (ht)”. When the material is cooled down, this absorbs water from the environment which induces another structural change: the structure closes, “low temperature (lt)”.^21^ These structural transformations considerably modify the characteristics of the pores and these are crucial for the loading and release of the molecules to be transported. Since it is required that any drug interacts relatively weakly with the structure of MIL-53(Al), such interaction does not completely close the pore.

Thus, MOF materials (particularly MIL-53(Al)) are suitable to host molecules with a biological or biochemical function. Herein, we explore the loading of sulfadiazine in MIL-53(Al) and its controlled released in local areas of the skin. A topical administration of antibiotic does not affect the cellular physiological process.^22^ Thus, we propose an evaluation *in vitro* of toxicity of MOF, assuming that Al^3+^ ions induce a certain neurotoxicity because of their interaction with DNA.^23,24^

## Results and discussion

### MOF and MOF–sulfadiazine systems

**Fig. 1:**
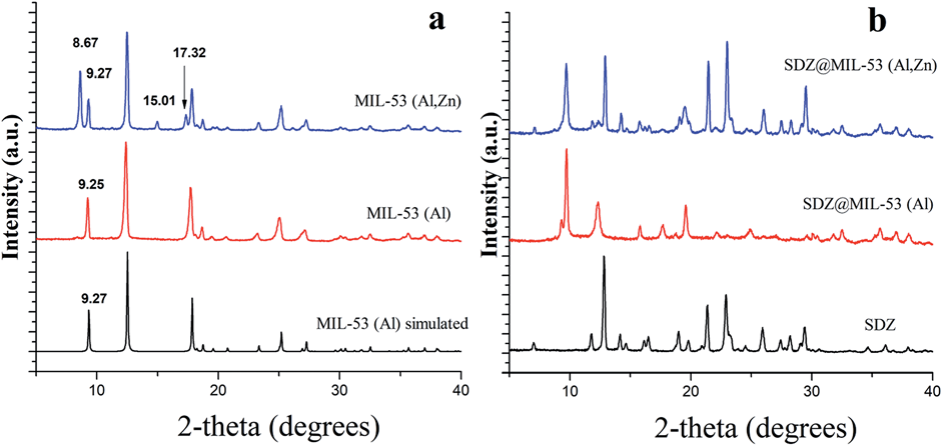
XRD patterns of sulfadiazine-free MOF materials (a) and loaded sulfadiazine–MOF materials (b).

Fig. 1a shows the XRD patterns of MIL-53(Al) and a modified MIL-53(Al) sample where a part of the Al(iii) metal centres was replaced by Zn(ii) (MIL-53-(Zn,Al)). The XRD pattern of both synthesized MOFs showed crystalline structures and these matched accordingly with previously reported XRD for MIL-53(Al), evidencing that the MOFs was successfully synthesised. When Zn(ii) is present in MOF structure, all of the MOF diffraction peaks appears at same position of that found for phase “lt” of MIL-53(Al). However, three additional diffraction peaks were observed at 17.32, 15.01 and 8.67 degrees which are attributable to phase “ht” of MIL-53(Al). The incomplete conversion between the two phases MIL-53(Al) was previously explained due to the presence of defects or residuals strain in the crystallites, hindering the complete conversion to the monoclinic phase.^25^ The ratio Zn(ii) : Al(iii) in (MIL-53-(Zn,Al) was 1.0 : 3.8 as measured by ICP-MS analysis.

**Fig. 2:**
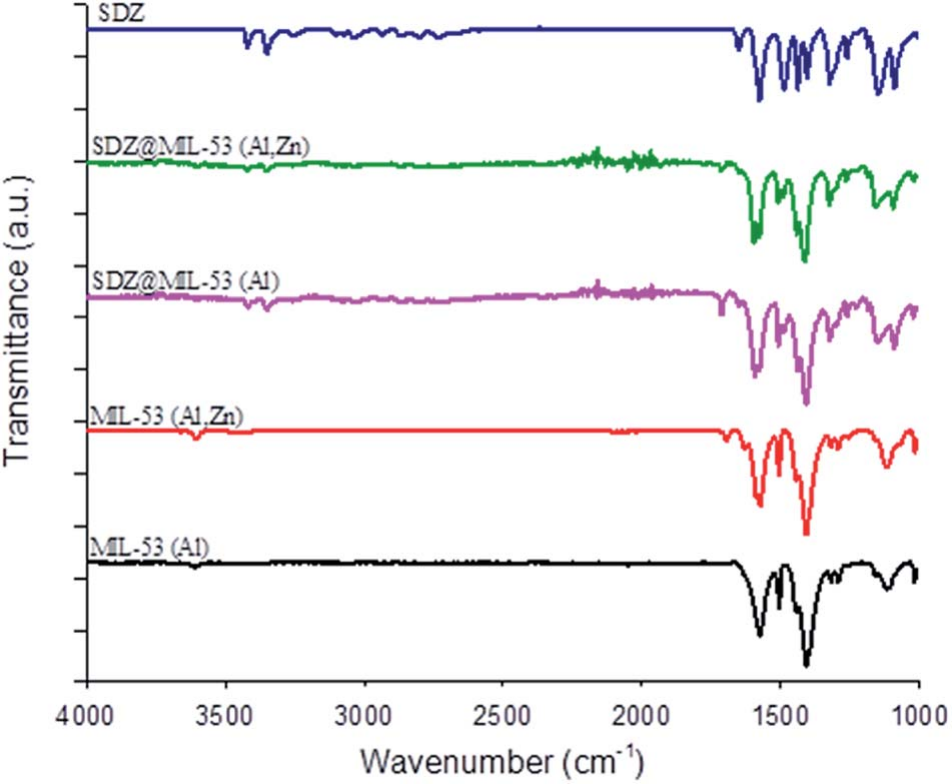
FTIR spectra of sulfadiazine-free MOF materials and loaded sulfadiazine–MOF materials.

After the loading of SDZ, modifications in the intensities on the ratio of the Bragg reflections (Fig. 1b), revealed a preferential orientation of crystallites forced during drug loading. Further, peaks highest intense changed their angle diffraction supporting that interatomic distances and bond angles in the MOF structure modified. Note that this was expected as the size of SDZ is close to the pore size of the MOF material and consequently, an interaction MOF–SDZ occurred. Besides, characteristic peaks of sulfadiazine were only detected in loaded SDZ–MIL-53(Al,Zn), which can be attributed to a partial encapsulation of SDZ in the MIL-53(Al,Zn) carrier. In line with this result, the FTIR spectra included in Fig. 2 show the presence of main absorption bands of sulfadiazine in MOF–SDZ materials. The IR spectra of MIL-53(Al) and MIL-53(Al,Zn) mainly showed the absorption bands due to benzene-carboxylates. For example the characteristic band at 1586 cm^−1^ was attributed to the C=O bonding in the carboxylates, and the band at 1411 cm^−1^ was from the aromatic carbon C–C vibrational mode. As the SDZ was loaded in MOF carriers, the bands at 1251 cm^−1^ appeared, corresponding to the stretching modes of aromatic carbon C–N bonding.^26,27^ Absorption bands characteristics of SDZ were also observed at 3420 cm^−1^ and 3349 cm^−1^ ascribed to N–H symmetric stretching mode and at 1317 cm^−1^ and 1150 cm^−1^ assigned to SO_2_ asymmetric stretching mode.

**Fig. 3:**
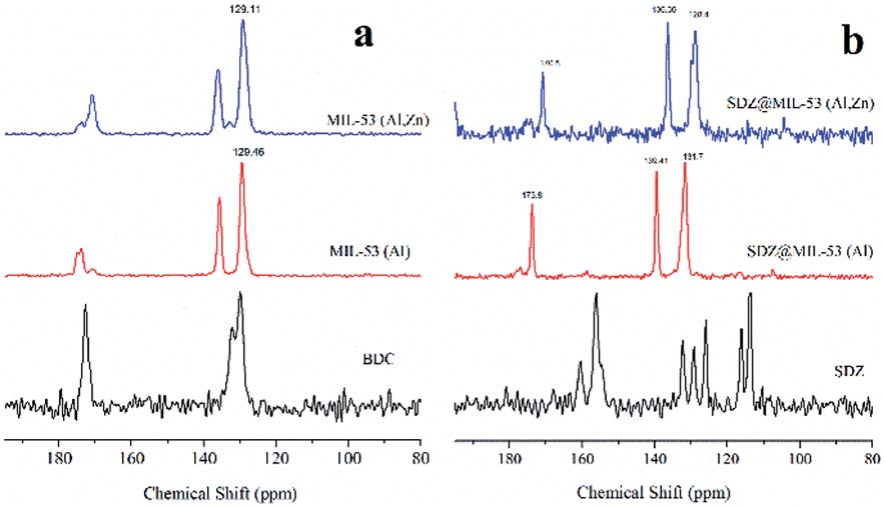
^13^C CP MAS NMR spectra of (a) sulfadiazine-free MOF materials (a) and loaded sulfadiazine–MOF materials (b).

Fig. 3a displays the ^13^C CP MAS NMR spectra of MIL-53(Al) and MIL-53(Al,Zn) without sulfadiazine. The signals between *δ* = 129 and 137 ppm are assigned to the aromatic carbons.^28^ Signals close to 170 ppm are attributable to the carbons of the carboxylate in both, the protonated and deprotonated forms. Note that the signal from the carboxylate is split. This can be explained due to the co-existence of the anionic form of the dicarboxylate from the framework and the acidic form present inside the pores. The presence of Zn(ii) does not change significantly the ^13^C CP MAS NMR spectra in line with stability of structure. Further, ^27^Al MAS NMR experiments (spectra not showed) confirmed that coordination of Al(iii) remains octahedral after the incorporation of Zn(ii).

^13^C CP MAS NMR spectra of both MOFs loaded with sulfadiazine are showed in Fig. 3b. The signals attributable to sulfadiazine (see the reference spectrum) are not undeniably observed in these spectra, only very low intense signals are present at 116 and 118 ppm. However, the loading of sulfadiazine considerably modifies the resonance peaks of the MOFs. The resonances due to aromatics carbons, shifted 4 ppm to weaker fields and the resonance due to carboxylic carbon shifted 1 ppm to stronger fields. The intensity of the signals close to 139 and 170 ppm is considerably increased. Thus, these results suggest that the two types of hydrogen atoms leading to cross-polarization are those of the C–H units and those of the adsorbed molecules inside pores. As the cross-polarization efficiency of the C–H in the free-sulfadiazine sample demonstrated to be sufficient to cross-polarize all the carbons, the gain of efficiency must be due to the additional, presence of sulfadiazine molecules. In summary, NMR results suggest that aromatic rings tend to stack due to the π–π interactions, as previously observed.^29–31^

The N_2_ adsorption–desorption isotherms displayed isotherms type I,^32^ which was associated with microporous materials. The Brunauer–Emmett–Teller (BET) surface area and pore volume of sulfadiazine-free and sulfadiazine-loaded MOFs are reported in Table 1. The MIL-53(Al) and MIL-53(Al,Zn) had specific surface areas of 1022.6 and 1099.3 m^2^ g^−1^, respectively. As the sulfadiazine was loaded at the MOF materials, the specific BET area decreased significantly. Taking into account the size of the pores of the MOFs and the kinetic diameter of the sulfadiazine molecules, it is reasonable to propose that the decrease in the surface area is due to the incorporation of sulfadiazine in the pores of the MOFs.

**Table 1:** Specific surface area and volume of pore of MOF and SDZ@MOF samples

Sample	*S*_BET_ [m^2^ g^−1^]	*V*_pore_ [cm^3^ g^−1^]
MIL 53(Al)	1022.6	234.9
MIL 53(Al,Zn)	1099.3	252.6
SDZ@MIL 53(Al)	671.7	154.3
SDZ@MIL 53(Al,Zn)	402.5	92.5

**Fig. 4:**
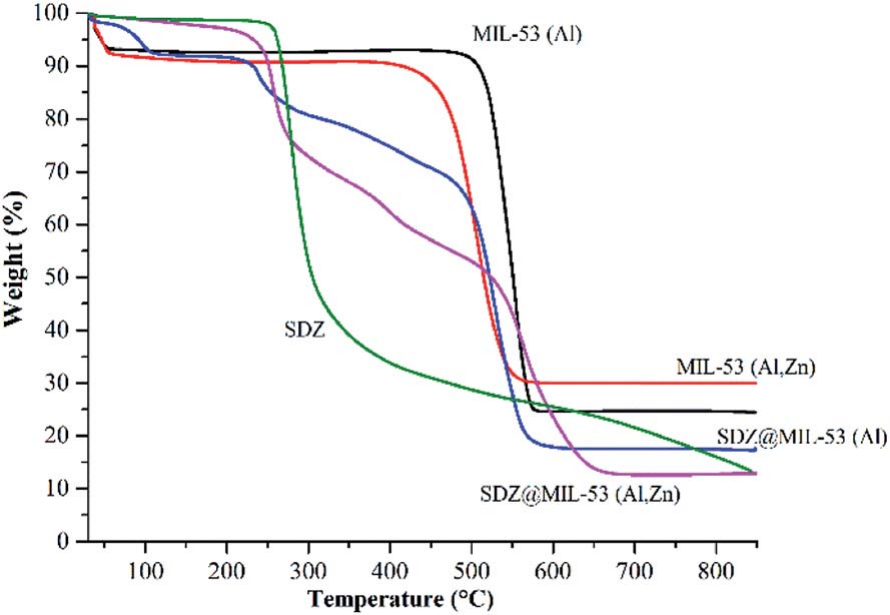
TGA curves of MOF and sulfadiazine@MOF materials. The curve of sulfadiazine is included as a reference.

The TG curves of the MOFs and SDZ@MOFs are shown in Fig. 4. The TG profile of sulfadiazine is also included as a reference. A first weight loss is observed for the free-sulfadiazine MOFs between 30 and 56 °C, this weight loss corresponded to 8.5 and 9.0% for MIL-53(Al) and MIL-53(Al,Zn), respectively and this step is attributable to desorption of water adsorbed within the MOF materials.

This weight loss is also observed in the sample SDZ@MIL-53(Al) but the step is shifted until 106 °C. In contrast, the first weight loss for SDZ@MIL-53(Zn,Al) corresponded only to 4% and occurs at relative high temperature (104–221 °C). Degradation of free-sulfadiazine samples occurs in a second step, samples MIL-53(Al) and MIL-53(Al,Zn) are decomposed between 468–581 °C and 394–562 °C, respectively. In addition to the decomposition step, in TG curves for SDZ@MOF another weight loss step was observed at 220 °C and corresponds to the sulfadiazine loss. According to this step, the amount of sulfadiazine was estimated and reported in Table 2.

**Table 2:** Sulfadiazine (%) loaded into the hosts MOFs

Sample	Sulfadiazine amount (%)	
	TGA	HPLC
SDZ@MIL 53(Al)	25	23.6
SDZ@MIL 53(Al,Zn)	40	38.9

### Sulfadiazine as guest in MOF hosts

The Table 2 reports the amount of sulfadiazine loaded on different MOFs. The loaded sulfadiazine amount is expressed as a percentage of the sulfadiazine retained by the MOFs from the total amount that was in contact with particles. Both techniques used to quantify the sulfadiazine confirm that the highest fraction of sulfadiazine was loaded in the MOF containing Zn(ii).

As the textural properties revealed, MIL-53(Al,Zn) was the material with the highest surface area before the sulfadiazine loading and also the material with the lowest surface area after SDZ incorporation. Thus, it is clear that surface area is a crucial parameter in order to incorporate the sulfadiazine into these MOF materials. Further, as previously mentioned, the interactions π–π also contribute to stabilization of SDZ into the MOFs.

### Sulfadiazine release from SDZ@MOF

#### In vitro

**Fig. 5:**
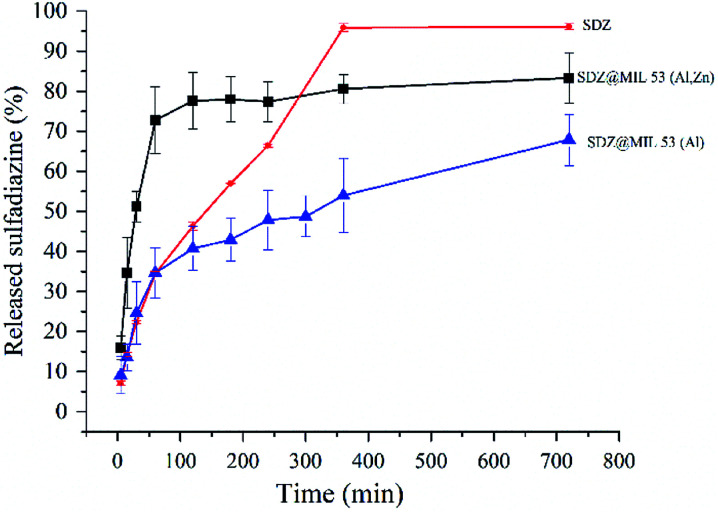
Release profile of sulfadiazine from sulfadiazine@MOF materials. Curve labelled SDZ refers to pure sulfadiazine permeation through the membranes.

The drug-release kinetics of SDZ@MOFs were assessed by *in vitro* studies using the dialysis membrane diffusion technique as (Experimental section). Fig. 5 shows that in both MOFs, two different regimes can be identified in the drug-release profile. For the SDZ@MIL-53(Al) and SDZ@MIL-53(Zn,Al) samples the first regime releases 35% of SDZ and 72%, respectively, within the first 2 h. This behaviour could be related to the presence of SDZ released from SDZ@MOFs, as well as to the drug outside of SDZ@MOFs. The second stage, the release process becomes slower, thus, the delivery of 28.2% of ZDZ occurred in a period of 2–12 hours in SDZ@MIL-53(Al) whereas only 10% of sulfadiazine is released for SDZ@MIL-53(Zn,Al). The comparison of the curves for pure sulfadiazine and release of SDZ from SDZ@MIL-53(Al,Zn) leads to the most interesting result: at short periods, a major release is achieved when sulfadiazine is incorporated into MIL-53(Al,Zn). The release curve for pure sulfadiazine is, indeed, the permeation of sulfadiazine trough the membrane and it is limited by the solubility of sulfadiazine which is indeed very low. However, the fact that the release increases with MIL-53(Zn,Al) suggests that adsorbed sulfadiazine could be released as an ionized molecule because of the high acidic p*K*_a_ (6.5) of sulfadiazine. Further, note that MIL-53(Zn,Al) is the carrier with the highest specific surface and after incorporation of SDZ is the material with lowest specific surface. Thus, if assumed that difference of specific area is the area occupied by the drug stacked through π–π SDZ–carrier interactions, this surface area occupied in MIL-53(Zn,Al) is 696.8 m^2^ g^−1^ and it should be enough to highly disperse SDZ and release it according to the profile of Fig. 5.

The *in vitro* drug release profile was fitted to different mathematical models such as the Higushi, Hixson–Crowell, Weibull and Korsmeyer–Peppas. Results were interpreted in the form of a graphical presentation and evaluated by the correlation coefficient. The highest degree on the correlation coefficient, determines the suitable mathematical model that follows certain drug release kinetics.^33^ In our case, it was found that the Korsmeyer–Peppas model^34^ showed higher degree of correlation coefficient than other models. Hence, sulfadiazine release profile from SDZ@MOF follows the type of diffusion suggested by model the Korsmeyer–Peppas. In this model the drug release follows the equation: *M*_*t*_/*M*_∞_ = *K*_kp_*t*^*n*^ being *M*_*t*_/*M*_∞_ a fraction of the drug released at time *t*. To match the results of release kinetics, a graph was plotted between log cumulative% drug release log(*M*_*t*_/*M*_∞_) *vs.* log time (log *t*). Hence, *n* value was estimated, which is used to characterize different release mechanisms. In our case, *n* was equal to 0.37 and 0.25 for the release results in SDZ@MIL-53(Al) and SDZ@MIL-53(Zn,Al), respectively. Thus these values suggest that the drug release from the SDZ–MOFs, proceeded according to a quasi-Fickian transport. In other words, the release mechanism was determined by diffusion, which occurred partially at the surface of SDZ–MOFs particles and partially within the pores of the MOFs.^35^

#### Ex vivo

An *ex vivo* permeation study was performed to determine the distribution of the drug throughout the stratum corneum, epidermis and dermis. The main target of this investigation was related to the ability of SDZ to passes through the skin and reach the blood circulation from SDZ@MIL-53(Al) and SDZ@MIL-53(Al,Zn).

**Fig. 6:**
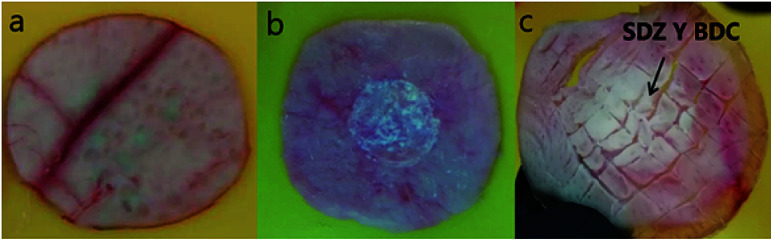
Dermis from pigskin (a); sulfadiazine in epidermis (b); SDZ and BDC in dermis (c).

The permeation of pure sulfadiazine suspended in 0.1 M PBS (pH 7.4) was practically absent. A high accumulation was observed in the surface layer of the stratum corneum, Fig. 6a. At the systemic level, the presence of SDZ was not determined because, in this case this occurred at a concentration lower than the detection limit (2 μg mL^−1^) of the validated chromatographic method for the quantification of the drug. The absent permeation was expected due to the insolubility of SDZ.

Similarly, the permeation of the MIL-53(Al) and MIL-53(Al,Zn) dispersed in PBS was not detected in the bloodstream, however accumulation in the epidermis and dermis is observed after performing cuts in the skin membrane and perform the extraction with 0.025 M NaOH, observing the presence of BDC (Fig. 6b and c). Interestingly, SDZ from the MOFs was systemic permeated after periods as long as 3 h. SDZ released from SDZ@MIL-53(Al) was detected after 180 min. After 300 min the presence of BDC was also detected.

While SDZ coming from SDZ@MIL-53(Al,Zn) permeated after 300 min and the presence of BDC was detected after 360 min. These conditions are favourable when permeation through the corneal extract and the epidermis is required, but not a systemic permeation, guaranteeing a local administration of an antibiotic used in prophylaxis and wound infections.

#### Cytotoxicity

**Fig. 7:**
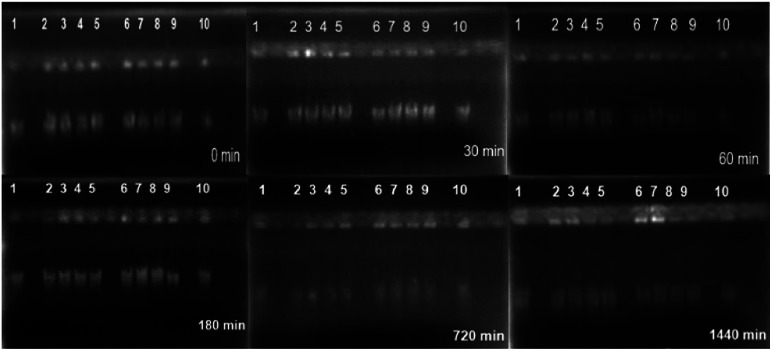
Image of DNA in agarose gel. ADN control (lanes 1 and 10); MIL-53(Al) (lanes 2 and 3); SDZ@MIL-53(Al) (lanes 4 and 5); MIL-53(Al,Zn) (lanes 6 and 7); SDZ@MIL-53(Al,Zn) (lanes 8 and 9).

Al^3+^ ions are neurotoxic agents and are involved in many neurochemical reactions because of disruption of DNA as a consequence of formation of DNA–Al^3+^ complexes.^23,24^Fig. 7 presents the electrophoretic analysis of systems MIL-53(Al) (lane 2 and 3), SDZ@MIL-53(Al) (lane 4 and 5), MIL-53(Al,Zn) (lane 6 and 7) and SDZ@MIL-53(Al,Zn) (lane 8 and 9) at concentrations of 1000 and 500 μg mL^−1^, after mixing with 20 μL of DNA at a concentration of 0.030 μg mL^−1^ at times of 0, 30, 60, 180, 720 and 1440 min. Same molecular weight is observed in lanes where MOFs were present if compared to a control solution of integral genomic material (lane 1 and 10), incubated at same treatment conditions meaning that Al^3+^ as a part of the MOF did not degrade DNA. The Zn^2+^ does not present any cytotoxicity. It is well known that Zn^2+^ plays important biological roles such as cofactor in metalloproteins. Close to 10% of human codifying proteins contain at least a Zn^2+^ site where the direct interaction with DNA is weak and do not lead to DNA damage.^24,36^ In other words, these MOFs and SDZ@MOF materials are not toxic for eukaryote cells.

## Conclusions

Replacement of 25% of Al(iii) by Zn(ii) in MIL 53(Al) leads to a mixture of phases low-temperature and high temperature of MIL 53(Al). Sulfadiazine was incorporated into pores of MIL-53(Al) and the zinc modified MIL-53(Al) version. The sulfadiazine is released *in vitro* from the sulfadiazine@MOF materials following a quasi-Fickian transport. The sulfadiazine release demonstrated to be faster for the MOF containing Zn(ii) in comparison to the free-zinc MOF. The presence of Zn(ii) enhances the solubility of sulfadiazine in aqueous media. The sulfadiazine and BDC, ligand of the MOF, permeate through the pig skin after periods as long as 3 hours. Remarkable, sulfadiazine@MOFs are not toxic against eukaryote cells.

## Experimental

### MOF synthesis

The following reagents were used without further purification. Terephthalic acid (BDC, 98%), aluminum nitrate nonahydrate (Al(NO_3_)_2_·9H_2_O, 99%), zinc acetate dihydrate (C_4_H_6_O_4_Zn·2H_2_O, 98%), zinc nitrate hexahydrate (Zn(NO_3_)_2_·6H_2_O, 98%), acetone (C_3_H_6_O, 98%) and ethanol (C_2_H_6_O, 95%) were purchased from Sigma. Acetonitrile grade HPLC (ACN 99.9%), water grade HPLC and glacial acetic acid were supplied by J.T. Baker.

The sample MIL-53(Al) was synthesized solvothermally by using a water solution in a Teflon-lined steel autoclave. A mixture of aluminium nitrate nonahydrate (35 mmol, 13.12 g), terephthalic acid (17.5 mmol, 2.88 g) and distilled water (50 mL) was incubated at 220 °C for 3 day. Then it was immersed in 50 mL of acetone for 30 min until a white powder precipitated. The white powder was dried at 100 °C and it was calcined at 400 °C for 12 h, and finally calcined a 330 °C for 24 h.

The Zn(ii)-doped MIL-53(Al) was synthesized as follows: Al(NO_3_)_2_·6H_2_O (17.5 mmol, 6.56 g), C_4_H_6_O_4_Zn·2H_2_O (17.5 mmol, 3.84 g) and BDC (17.5 mmol, 2.88 g) were dissolved in water (50 mL). The mixture was stirred until obtaining the homogeneous solution, followed by transfer into a 100 mL Teflon-lined autoclave and the temperature increased to 220 °C. After 3 days, the white powder was washed several times with acetone and then dried at 100 °C.

### Characterization methods

X-ray powder diffraction (XRD) patterns were obtained using a D8 Advance (Bruker), with a copper Kα radiation source. ^13^C CP MAS NMR spectra were obtained at a Larmor frequency of 75.4 MHz using a Bruker Avance 300 spectrometer equipped with a 4 mm cross-polarization (CP) MAS probe. The samples were spun at a rate of 5 kHz. Spectra were recorded using a contact time of 5 ms and π/2 pulses of 5 μs. The chemical shifts were referenced to TMS.

Textural properties of MOFs were characterized by N_2_ adsorption–desorption isotherms, which were obtained at −196 °C in a BELSORP-mini II instrument.

### Incorporation of SDZ to MOFs

The MOF materials were dried at 200 °C for 12 h before suspended in the SDZ solution. A dried sample of 50 mg of each MOF was separately weighed and mixed with the SDZ solution in 10 mL glass containers. After sealing the container, the mixture was stirred (at 700 rpm) for 48 h at room temperature by a magnetic stirrer. The supernatant was collected.

The MOF loaded with SDZ (SDZ@MOFs) was immediately washed with 30 mL of acetone and centrifuged to remove SDZ adsorbed on the outer surface of MOFs. Samples containing sulfadiazine were labelled as SDZ@MIL 53(Al) and SDZ@MIL 53(Al,Zn). After loading SDZ@MOF material was dried overnight at 100 °C in an oven.

SDZ@MOF were analysed by HPLC to determine the amount total of SDZ. The SDZ calibration assay was analysed by high-performance liquid chromatography-UV (HPLC-UV) (Agilent 10 000) at a range of SDZ concentration between 10 and 200 μg mL^−1^ in sodium hydroxide [0.025 N]. The mobile phase was a mixture of water, ACN and glacial acetic acid (87, 12 and 1% respectively).

### SDZ release profiles

For *in vitro* experiments a known quantity of system MOF loaded with SDZ was immersed into 10 mL of preheated dissolution medium PBS (0.1 M) at pH 7.4 in sealed 30 mL capacity glass vials maintained at 37 ± 1 °C with a constant stirring at a rate of around 300 rpm.

An aliquot of 100 μL was withdrawn at different times and replaced with the same volume of fresh dissolution medium. The aliquots were filtered by 0.2 μm syringe filter and analysed using HPLC. A correction of the SDZ amount in dissolution medium extracted was calculated regarding the SDZ lost in each aliquot.

The *ex vivo* delivery proceeded as follows: the pig skin, obtained from pig ears, was cut into circular sections of 3 cm in diameter. The excess fat was removed. The samples were washed with saline and subsequently with 0.1 M PBS (pH 7.4) solutions. 6 Franz cells were used where the temperature was maintained constant at 37 ± 0.5 °C and the diffusion area was 7.07 cm^2^.

The pig skin was placed in Franz's cells and the stratum corneum remained in contact with the donor cell compartment, with the dermis towards the recipient's compartment which was filled with 30 mL of 0.1 M PBS solution (pH 7.4) and kept under constant stirring at 350 rpm.

1 mL of a suspension of the SDZ@MOF was transferred onto the donor cell containing 8 mL of PBS (0.1 M, pH 7.4) and transferred onto the donor cell at a concentration of 2 mg mL^−1^, a stirring of 350 rpm is maintained and a sampling of 1 mL is performed with replacement of medium at period times of 0, 15, 30, 60, 180, 240, 300 and 360 min. The samples are quantified by HPLC method as the same procedure as the release profiles.

At the end of the test, the skin samples were extracted from Franz cells and fragmented into small pieces with a surgical scalpel to extract the sulfadiazine from the layers with 30 mL of SDZ in 0.025 M NaOH. The extract was centrifuged at 2000 rpm for 10 min and analysed by UV-vis HPLC.

### DNA degradation

DNA of leucocytes extraction and quantification proceeded as follows: blood was drawn through venous puncture with Vacutainer™ tubes from EDTA/K2 and the rapid extraction protocol of the Wizard® Promega Genomic DNA Purification Kit was performed. 2 μL of DNA solution was taken and quantified with a dark field microcell with an Eppendorf® brand biophotometer.

For MOF interaction treatments with DNA, a standard 5× TBE buffer solution was prepared. The pH was adjusted to 8.3 and a 1 : 10 dilution was performed to obtain a working solution of 0.5× TBE. Subsequently, 20 μL of DNA solution was mixed with 20 μL of MOF suspended suspension in TBE 0.5 varying concentration of suspension at 1000 and 500 μg mL^−1^ and then samples incubated at 37 °C. Sampling of 2 μL was performed at different periods, and mixed with 2 μL of loading buffer to deposit them in the wells of the agarose gel at 1.5%. They were then placed in an electrophoresis chamber with a solution of 100 mL TBE 0.5× at conditions of 100 V for 35 min. At the end of the time it was revealed with 10 mL of a solution of ethidium bromide (2 mg mL^−1^) in 100 mL of 0.5× TBE and placed in the Bio-Rad Universal Hood II photodocumentor – Software Quantity One.

## Conflicts of interest

There are no conflicts to declare.

## Supplementary Material
